# Diagnostic Value of Serum 25-Hydroxyvitamin D Levels in Predicting Poor Glycemic Control Among Children with Type 1 Diabetes Mellitus: A ROC Curve and Decision Curve Analysis

**DOI:** 10.3390/diagnostics16111661

**Published:** 2026-05-28

**Authors:** Youssef A. Alqahtani, Ayed A. Shati, Ayoub A. Alshaikh, Ashwag Asiri, Saleh M. Alqahtani, Nada Hamzah Albarqi, Fatmah Qasim Shamakhi, Ramy Mohamed Ghazy

**Affiliations:** 1 Department of Child Health, College of Medicine, King Khalid University, Abha 61421, Saudi Arabia; youssef98118911@gmail.com (Y.A.A.); shatiayed@gmail.com (A.A.S.); asalasiri@kku.edu.sa (A.A.); smuadi@kku.edu.sa (S.M.A.); 2 Department of Family and Community Medicine, College of Medicine, King Khalid University, Abha 61421, Saudi Arabia; 3 Asser Health Cluster, Ministry of Health Saudi Arabia, Abha 62461, Saudi Arabiafatmah.sh.1997@gmail.com (F.Q.S.); 4 Tropical Health Department, High Institute of Public Health, Alexandria University, Alexandria 21526, Egypt; 5 Health and Medical Research Centre, King Khalid University, Abha 61421, Saudi Arabia

**Keywords:** vitamin D deficiency, type 1 diabetes mellitus, glycemic control, HbA1c, pediatrics, ROC curve, decision curve analysis

## Abstract

**Background/Objectives**: Children and adolescents with type 1 diabetes mellitus (T1DM) may be particularly vulnerable to vitamin D deficiency; however, its association with glycemic control remains incompletely understood. This study aimed to determine the prevalence of vitamin D deficiency among children and adolescents with T1DM and to evaluate its relationship with glycemic control. **Methods**: This cross-sectional study enrolled individuals aged 1–18 years diagnosed with T1DM. Serum 25-hydroxyvitamin D concentrations were assessed, with deficiency defined as <20 ng/mL, insufficiency as 20–29 ng/mL, and sufficiency as ≥30 ng/mL. Glycemic control was defined as HbA1c < 9.0% (controlled) versus ≥9.0% (poor control). Multivariable logistic regression was performed to assess the independent association of 25-hydroxyvitamin D with glycemic control. Receiver operating characteristic (ROC) curve analysis and decision curve analysis (DCA) were conducted to evaluate the diagnostic and potential clinical utility of 25-hydroxyvitamin D levels. **Results**: A total of 266 participants were included; their median age was 13.0 years (IQR: 10.0–15.0), with a slight male predominance (56.8%). Overall, 64.3% of patients had suboptimal 25-hydroxyvitamin D status, including 30.1% with deficiency and 34.2% with insufficiency. Patients with poor glycemic control had significantly lower 25-hydroxyvitamin D levels compared to those with controlled diabetes (21.0 ng/mL [IQR: 17.0–26.0] vs. 35.0 ng/mL [IQR: 28.0–45.0], *p* < 0.001). A strong negative correlation was observed between HbA1c and 25-hydroxyvitamin D levels (Spearman’s ρ = −0.651, *p* < 0.001). Vitamin D insufficiency was significantly associated with poor glycemic control (aOR = 4.74, 95% CI: 2.48–9.32), while vitamin D deficiency was associated with substantially greater odds (aOR = 40.59, 95% CI: 15.55–129.23). The optimal cut-off for predicting poor control was 26.5 ng/mL, achieving a sensitivity of 75.5% and specificity of 82.2%. Decision curve analysis confirmed that the 25-hydroxyvitamin D model provided superior net benefit compared to treat-all and treat-none strategies across a threshold probability range of 32–85%. **Conclusions**: Vitamin D deficiency and insufficiency are highly prevalent among children and adolescents with T1DM. Lower 25-hydroxyvitamin D levels are independently associated with poorer glycemic control. Serum 25-hydroxyvitamin D demonstrates good diagnostic accuracy and potential clinical utility for risk stratification. Screening for 25-hydroxyvitamin D status and consideration of supplementation may serve as an adjunctive strategy to support metabolic management in this population.

## 1. Introduction

Vitamins are essential for the body’s immunopathological and physiological functions. Specifically, vitamin D, a fat-soluble vitamin, plays a crucial role in a wide range of biological and physiological processes [1]. It is primarily synthesized endogenously in the skin. Vitamin D has two major forms: ergocalciferol (D2) and cholecalciferol (D3) [2]. Following sunlight exposure, vitamin D is produced from 7-dehydrocholesterol in the skin [3]. Once produced or ingested, vitamin D undergoes hepatic hydroxylation to form 25-hydroxyvitamin D, the principal circulating form and the most reliable biomarker used to assess vitamin D status in clinical practice [4]. Vitamin D synthesis is influenced by factors such as time of day, season, geographic location, skin pigmentation, and sunscreen use. Risk factors for vitamin D deficiency include limited sun exposure, darker skin, aging, obesity, low dietary intake, certain medications, malabsorption, and liver or kidney disorders [5].

Vitamin D deficiency remains a major global public health concern [6]. Between 2000 and 2022, the global prevalence of vitamin D deficiency was estimated at 45% (95% CI: 37–54%), with substantial geographic variation ranging from 57% (95% CI: 45–70%) in regions at 60–80° N latitude to 18% (95% CI: 11–27%) in regions at 20–60° N. Vitamin D deficiency has been associated with a wide spectrum of chronic conditions, including cardiovascular disease, inflammatory disorders, and cancer [7,8,9]. Beyond its classical role in calcium homeostasis and bone metabolism, vitamin D has an immunomodulatory function as it regulates innate and adaptive immune responses [10]. Serum 25-hydroxyvitamin D concentration levels below 20 ng/mL have been strongly associated with adverse health outcomes, whereas concentrations above 20–30 ng/mL are generally considered beneficial for overall health [11].

Type 1 diabetes mellitus (T1DM) is the most common form of diabetes in children and adolescents [12]. T1DM can lead to acute complications such as diabetic ketoacidosis (DKA), which carries high morbidity and mortality risks if not promptly diagnosed and treated.T1DM can lead to acute complications such as diabetic ketoacidosis (DKA), which carries high morbidity and mortality risks if not promptly diagnosed and treated [13]. A high prevalence of vitamin D deficiency has been consistently reported in individuals with T1DM. They tend to have lower serum 25-hydroxyvitamin D levels compared to healthy controls [14,15]. The pooled analysis showed that 46% (95% CI: 34–58%) of children and adolescents with T1DM had vitamin D deficiency [16,17,18]. Emerging evidence suggests that vitamin D may influence insulin secretion in pancreatic tissue, contribute to the prevention of T1DM and its complications, play a role in glucose metabolism, and affect disease pathophysiology [19]. An inverse relationship between serum 25-hydroxyvitamin D and glycated hemoglobin (HbA1c) has been reported [20]. The widespread distribution of 25-hydroxyvitamin D receptors (VDRs) across multiple tissues, including immune cells such as antigen-presenting cells, activated T and B lymphocytes, and pancreatic β-cells, supports its role in immune regulation and pancreatic function [21]. Vitamin D functions as a steroid hormone with pleiotropic effects on immune regulation and metabolic homeostasis. Its activity is influenced by estrogen status, with potential protective effects in diabetic kidney disease (DKD) [22,23]. Moreover, vitamin D supplementation has been shown to reduce proteinuria and slow the progression of kidney disease [24]. However, interventional findings remain inconsistent. For example, supplementation with cholecalciferol (2000 IU/day for 12 weeks) effectively corrects vitamin D deficiency in children and adolescents with T1DM, but does not consistently improve glycemic control, although post-supplementation vitamin D deficiency levels have been inversely associated with insulin requirements and body mass index (BMI) [25].

Saudi Arabia ranks ninth globally for the number of T1DM cases with approximately 46,000 children and adolescents affected [26]. Despite evidence linking vitamin D deficiency to T1DM, no study has yet established a serum 25-hydroxyvitamin D threshold for predicting poor glycemic control in children and adolescents with T1DM using combined ROC and decision curve analysis, particularly in high-prevalence settings such as Saudi Arabia. This study hypothesized that vitamin D deficiency is highly prevalent among children and adolescents with T1DM and that vitamin D deficiency is associated with poorer glycemic control in this population. Accordingly, the study aimed to determine the prevalence of vitamin D deficiency in children and adolescents with T1DM and to evaluate its association with glycemic control.

## 2. Materials and Methods

### 2.1. Study Design

This cross-sectional study analyzed data collected over a five-year period (2021–2025) for children and adolescents diagnosed with T1DM at King Khalid University Medical City and Abha Maternity and Children Hospital. Data collection was conducted between July 2024 and December 2024.

### 2.2. Study Population

All patients aged 1–18 years with a confirmed diagnosis of T1DM were included. Patients with incomplete demographic data or medical records were excluded. Additionally, individuals receiving vitamin D supplementation or medications known to affect vitamin D metabolism, including anticonvulsants, glucocorticoids, antifungals, and antiretroviral drugs, were also excluded.

### 2.3. Study Variables

The dependent variable was glycemic control, assessed by HbA1c levels. The primary independent variable was serum 25-hydroxyvitamin D level (ng/mL). Additional independent variables included demographic characteristics (age and sex), clinical characteristics (diabetes duration and time in range percentage), anthropometric measurements (BMI in kg/m^2^), and dietary pattern (balanced diet: yes or no).

### 2.4. Study Outcomes

The primary outcome was 25-hydroxyvitamin D status, categorized as deficiency, insufficiency, or sufficiency. The secondary outcome was the association between 25-hydroxyvitamin D status and glycemic control.

### 2.5. Data Collection

Demographic and clinical data were extracted from electronic medical records, including age (age and age at diagnosis), sex, diabetes duration, HbA1c level, time in range (from continuous glucose monitoring), anthropometric measurements (height, weight, and body mass index), serum 25-hydroxyvitamin D levels, and dietary information (balanced diet).

### 2.6. Operational Definitions

Vitamin D status: Vitamin D deficiency was defined as serum 25-hydroxyvitamin D < 20 ng/mL, insufficiency as 20–29 ng/mL, and sufficiency as ≥30 ng/mL, according to the Endocrine Society guidelines [27].

Glycemic control: Glycemic control was initially categorized according to American Diabetes Association (ADA) pediatric guidelines as optimal (HbA1c < 7.0%), acceptable (HbA1c 7.0–7.5%), suboptimal (HbA1c 7.5–9.0%), and poor (HbA1c ≥ 9.0%) [28]. For analytical purposes, these categories were combined into two groups: HbA1c < 9.0% and HbA1c ≥ 9.0%. An HbA1c ≥ 9.0% indicates persistent hyperglycemia, which is independently associated with a higher likelihood of long-term microvascular complications, including retinopathy, nephropathy, and neuropathy in children and adolescents, and represents a clinical risk condition for DKA, particularly when insulin doses are missed or during episodes of intercurrent illness [4,28].

Diabetes duration in years: Diabetes duration was determined in years, measured from the date of diagnosis to the date on which data were collected. For categorical analyses, participants were classified as new onset (<2 years), intermediate duration (2–5 years), or long-standing (>5 years).

Time in Range (TIR): TIR was defined as the proportion of time during which glucose values were within the target interval of 70–180 mg/dL. TIR was evaluated using the FreStyle Libre continuous glucose monitoring (CGM) system. All patients were on GCM. In accordance with international consensus guidelines on CGM metrics, TIR was classified as good when it was ≥70% [29]. Values below 70% were further categorized as fair (50–69%) or poor (<50%).

BMI classification: BMI was calculated as weight in kilograms divided by height in meters squared. BMI percentiles were determined using the Saudi age- and sex-specific growth charts. Categories were defined as underweight (BMI < 5th percentile), healthy weight (BMI 5th to <85th percentile), overweight (BMI 85th to <95th percentile), and obese (BMI ≥ 95th percentile) [30].

### 2.7. Laboratory Measurements

HbA1c measurement: Venous whole blood was collected into dipotassium EDTA (K_2_EDTA) tubes. HbA1c levels were quantified using ion-exchange high-performance liquid chromatography (HPLC) on a Tosoh G8 HPLC Analyzer (Tosoh Bioscience, Tokyo, Japan).

25-Hydroxyvitamin D measurement: Venous blood was collected into plain serum tubes. After clotting and centrifugation, serum 25-hydroxyvitamin D levels were measured using electrochemiluminescence immunoassay (ECLIA) on a MAGLUMI series chemiluminescence immunoassay analyzer (Shenzhen New Industries Biomedical Engineering Co., Ltd., Shenzhen, China). The method was standardized against NIST SRM 972a. The chemiluminescence reaction was measured as relative light units (RLUs), which are inversely proportional to the concentration of 25-Hydroxyvitamin D in the sample.

### 2.8. Ethical Approval

This study was conducted in accordance with the Declaration of Helsinki. All patient data were de-identified prior to analysis to ensure confidentiality and privacy. The study protocol was reviewed and approved by the Institutional Review Board (IRB) of King Khalid University (IRB No. KKU-16-2025-13, approved on 19 May 2025). The requirement for informed consent was waived by the IRB due to the retrospective nature of the study and the use of de-identified medical records.

### 2.9. Statistical Analysis

Statistical analyses were performed using R software (version 4.3.3, R Foundation for Statistical Computing, Vienna, Austria). A two-tailed *p*-value < 0.05 was considered statistically significant. The following R packages were utilized: dplyr and tidyr for data handling, ggplot2 for visualization, pROC for Receiver operating characteristic (ROC) curve analysis, and openxlsx and flextable for table export. Continuous variables were presented as medians with interquartile ranges (IQRs) after normality assessment using the Shapiro–Wilk test. Categorical variables were presented as frequencies and percentages. The association between vitamin D deficiency and categorical variables (gender, age group, glycemic control category, BMI category, diabetes duration) was assessed using the Chi-square test or Fisher’s exact test where appropriate. For continuous variables (age, HbA1c, diabetes duration, BMI), the Mann–Whitney U test was used to compare means between deficient and non-deficient groups. Spearman correlation coefficients were calculated to examine the correlation between numerical variables HbA1c and 25-hydroxyvitamin D levels. Correlation strength was interpreted as follows: weak (|r| < 0.3), moderate (0.3 ≤ |r| < 0.5), strong (0.5 ≤ |r| < 0.7), and very strong (|r| ≥ 0.7).

Binary logistic regression was performed to identify factors associated with HbA1c ≥ 9.0%. Univariate analysis was first conducted for each independent variable, including age (continuous, years), gender (categorical, female as reference), diabetes duration (continuous, years), 25-hydroxyvitamin D status (deficient, insufficient, and sufficient as reference), and body mass index (continuous, kg/m^2^). Variables with *p* < 0.20 in univariate analysis, along with clinically relevant variables, were entered into a multivariable logistic regression model using a forced-entry approach to adjust for potential confounders. Model fit was assessed using the Hosmer–Lemeshow goodness-of-fit test (*p* > 0.05 indicating acceptable calibration). Multicollinearity was assessed using variance inflation factors (VIF), with values <5 considered acceptable. Results are reported as odds ratios (OR) with 95% confidence intervals (CI) and two-tailed *p*-values, with statistical significance set at *p* < 0.05.

A ROC curve analysis was conducted to assess how well serum 25-hydroxyvitamin D levels distinguish between patients with HbA1c ≥ 9.0% and those with HbA1c < 9%. The area under the curve (AUC) was calculated with 95% confidence intervals (CIs) using DeLong’s method. The optimal clinical threshold for 25-hydroxyvitamin D was determined using Youden’s index (J = sensitivity + specificity − 1). At this threshold, sensitivity, specificity, positive predictive value (PPV), and negative predictive value (NPV) were calculated. AUC > 0.80 was considered good discrimination. To assess the practical application of the model, decision curve analysis (DCA) was conducted. Net benefit was calculated across a range of threshold probabilities (0–100%) to compare the 25-hydroxyvitamin D-based strategy against “treat-all” and “treat-none” strategies.

### 2.10. Data Quality Check

All collected data were independently reviewed to ensure accuracy and consistency. A second reviewer cross-checked a subset of the dataset against the original medical records and laboratory reports to verify data entry correctness. Discrepancies were resolved through reexamination of source documents and consensus between investigators. Data quality was assessed through systematic checks for completeness, range validity, and consistency. Outliers and implausible values were reviewed and verified against source records. Missing data were handled using complete-case analysis, as the proportion of missing data for key variables was less than 5%.

## 3. Results

Table 1 summarizes the demographic and clinical characteristics of the study population (*N* = 266). The median age was 13.0 years (IQR: 10.0–15.0), with the largest proportion aged 10–14 years (40.2%) and a slight male predominance (56.8%). The median duration of diabetes was 2.0 years [IQR: 1.0–4.0], with most participants classified as having intermediate disease duration (61.3%), followed by new-onset diabetes (32.7%). Overall glycemic control was suboptimal, with a median HbA1c of 9.7% (IQR: 7.8–11.4], and nearly three-fifths (59.8%) had poor glycemic control (HbA1c ≥ 9.0%). Similarly, 59.8% had poor TIR, with a median TIR of 40% [IQR: 0.3–0.7]. Regarding nutritional status, the median BMI was 18.9 kg/m^2^ [IQR: 17.0–22.3]. Most patients had normal weight (67.7%), although a notable proportion were underweight (3.8%), with smaller proportions classified as overweight (17.3%) or obese (11.3%).

**Figure 1:**
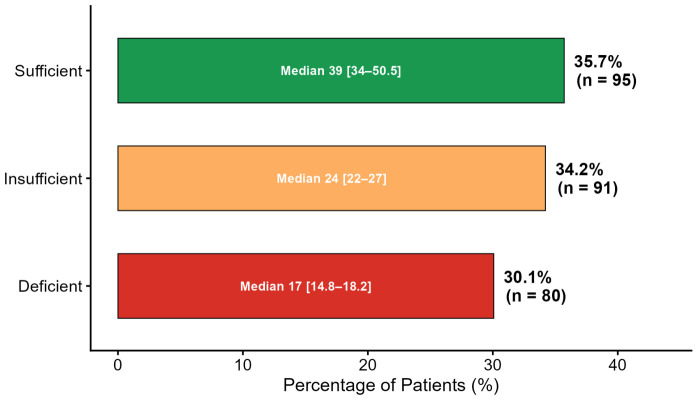
25-hydroxyvitamin D status distribution with median (IQR) levels. IQR: Interquartile range. *N* = 266 patients.

Among the 266 children and adolescents, 80 patients (30.1%) were vitamin D deficient (median level of 25-hydroxyvitamin D: 17 ng/mL (IQR: 14.8–18.2), 91 (34.2%) had insufficient levels with a median of 24 ng/mL (IQR: 22.0–27.0), and 95 patients (35.7%) had sufficient levels (median: 39 ng/mL (IQR: 34.0–50.5); see Figure 1.

A statistically significant association was observed between age group and vitamin D status (*p* = 0.032). The highest proportion of vitamin D sufficiency was found among participants aged 5–9 years (47.1%), followed by those aged 15–18 years (41.5%). In contrast, participants aged 10–14 years demonstrated the lowest sufficiency rate (26.2%) and the highest prevalence of suboptimal vitamin D status (73.8%). A statistically significant association was also identified between weight status and vitamin D status (*p* = 0.001). Overweight/obese participants showed the highest proportion of vitamin D sufficiency (88.9%), whereas participants with normal weight exhibited lower sufficiency rates (33.3%). Underweight participants had a predominance of suboptimal vitamin D levels (57.1%).

Balanced diet was significantly associated with vitamin D status (*p* = 0.006). Participants who did not follow a balanced diet had markedly higher rates of suboptimal vitamin D levels (86.1%) and very low sufficiency rates (13.9%) compared with those reporting a balanced diet (sufficiency: 39.1%). Regarding gender, no statistically significant association was observed with vitamin D status (*p* = 0.090); however, males demonstrated a higher proportion of vitamin D sufficiency (40.4%) compared with females (29.6%), while females showed a greater prevalence of suboptimal vitamin D levels (70.4% vs. 59.6%). No statistically significant association was identified between diabetes onset age and vitamin D status (*p* = 0.280). BMI category was not significantly associated with vitamin D status (*p* = 0.222). Nevertheless, obese participants demonstrated relatively higher vitamin D sufficiency (50.0%) compared with underweight (30.0%) and normal-weight participants (32.2%). Similarly, no statistically significant relationship was observed between height status and vitamin D status (*p* = 0.231). However, participants with short stature had the highest proportion of suboptimal vitamin D levels (90.0%) and the lowest sufficiency rate (10.0%) compared with those of normal stature or tall stature (Table 2).

**Figure 2:**
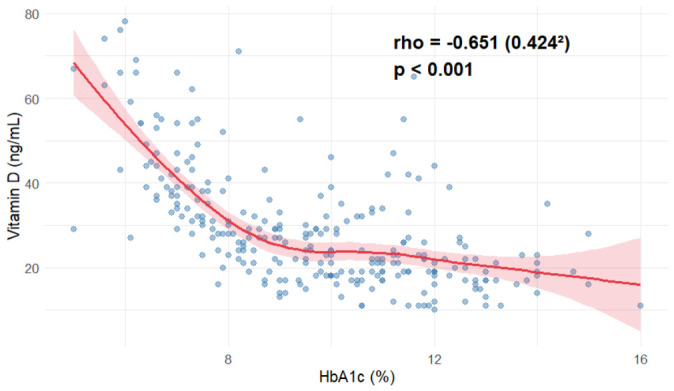
Correlation Correlation between HbA1c and 25-hydroxyvitamin D Levels in T1DM Patients.

A statistically significant strong negative correlation was observed between HbA1c and 25-hydroxyvitamin D levels (Spearman’s ρ = −0.651, *p* < 0.001). Lower 25-hydroxyvitamin D levels were associated with poorer glycemic control, reflected by higher HbA1c values. Bootstrap analysis further confirmed the robustness of this association (95% CI: −0.724 to −0.567). The squared correlation coefficient (ρ^2^ = 0.424) indicates that approximately 42.4% of the variability in the ranked relationship between HbA1c and 25-hydroxyvitamin D can be explained by their monotonic association; see Figure 2.

Of the participants studied, 159 (59.8%) exhibited poor glycemic control (HbA1c ≥ 9%). Serum 25-hydroxyvitamin D demonstrated a strong and statistically significant association with glycemic control. Median 25-hydroxyvitamin D levels were significantly higher in the group with HbA1C < 9% 35.0 [25–45] ng/mL compared with the HbA1c ≥ 9.0% group 21 [17–26] ng/mL, *p* < 0.001. Vitamin D deficiency was markedly more frequent among patients in the HbA1c ≥ 9.0% group (93.8% vs. 6.2%, *p* < 0.001), while vitamin D sufficiency was more common in the HbA1C < 9% group (73.7% vs. 26.3%). BMI did not show a statistically significant association with glycemic control. Median BMI was comparable between the two groups—19.0 [17.6–22.5] vs. 18.3 [16–22] kg/m^2^, *p* = 0.198—and no significant differences were observed across BMI categories (*p* = 0.645). Participants reporting a balanced diet were more likely to have HbA1c < 9% (45.2% vs. 54.8%), whereas those without a balanced diet were predominantly in the HbA1c ≥ 9.0% group (91.7% vs. 8.3%, *p* < 0.001). In contrast, demographic characteristics were not significantly associated with glycemic control. Median age was similar between groups—13.0 [10.0–15.0] vs. 13.0 [9.5–15.0] years, *p* = 0.507—as was age at onset: 10.0 [7.0–12.0] vs. 10.0 [6.5–12.0] years, *p* = 0.458. Sex distribution did not differ significantly between groups (*p* = 0.146). Similarly, diabetes duration showed no significant association with glycemic control. Median duration was identical in both group—2.0 [1.0–4.0] vs. 2.0 [1.0–3.0] years, *p* = 0.714—and categorical duration (new onset, intermediate, and long-standing) was also not statistically significant (*p* = 0.956); see Table 3.

The multivariable logistic regression model demonstrated acceptable overall performance and calibration. McFadden’s pseudo-R^2^ was 0.284, indicating moderate-to-strong explanatory power. Additional pseudo-R^2^ measures were consistent with this finding (Cox & Snell R^2^ = 0.312; Nagelkerke R^2^ = 0.425). Model calibration was satisfactory, as evidenced by a non-significant Hosmer–Lemeshow goodness-of-fit test (χ^2^ = 8.46, *p* = 0.389), suggesting no significant discrepancy between observed and predicted outcomes. Information criteria were as follows: log-likelihood = −156.32, AIC = 326.64, and BIC = 345.28.

**Figure 3:**
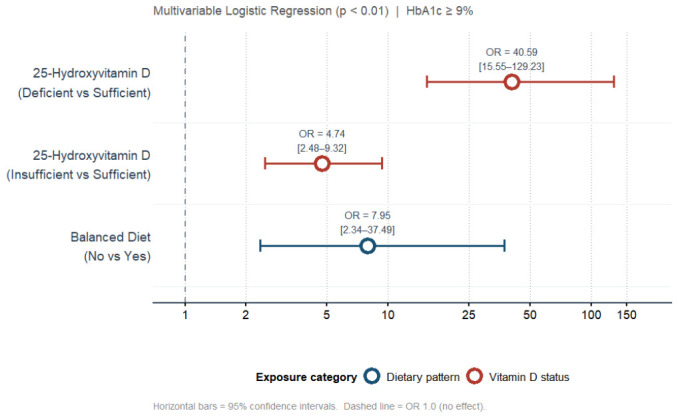
Multivariable logistic regression: factors associated with poor glycemic control.

Vitamin D deficiency was the strongest independent predictor of poor glycemic control (HbA1c ≥ 9%), with substantially increased odds compared with sufficient vitamin D status (aOR = 40.59, 95% CI: 15.55–129.23). Vitamin D insufficiency was also significantly associated with poor glycemic control (aOR = 4.74, 95% CI: 2.48–9.32). Additionally, absence of a balanced diet was independently associated with higher odds of poor glycemic control (aOR = 7.95, 95% CI: 2.34–37.49). In contrast, sex (aOR = 0.85, 95% CI: 0.45–1.59), age (aOR = 0.95, 95% CI: 0.86–1.04), diabetes duration (aOR = 0.96, 95% CI: 0.79–1.19), and BMI (OR = 0.98, 95% CI: 0.91–1.06) were not significantly associated with poor glycemic control, as their confidence intervals included the null value (Figure 3).

**Figure 4:**
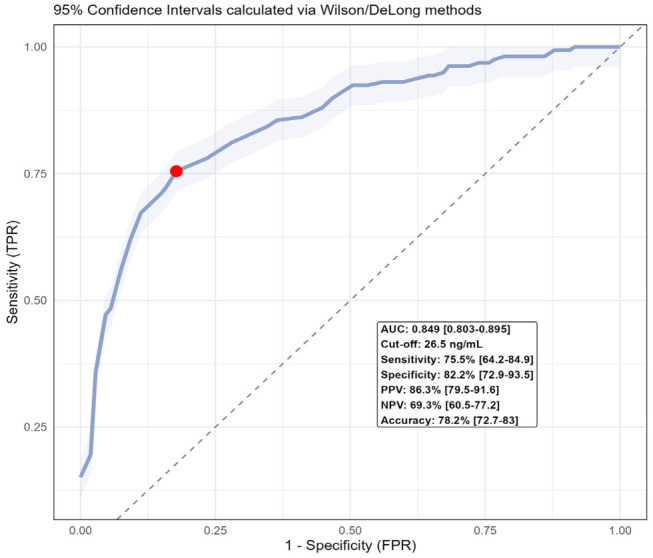
Receiver Operating Characteristic (ROC) curve showing the discriminative power of 25-hydroxyvitamin D.

Serum 25-hydroxyvitamin D demonstrated good discriminative ability for predicting poor glycemic control with an AUC of 0.849 (95% CI: 0.803–0.895, *p* < 0.0001). The optimal cut-off for predicting poor control was identified at 26.5 ng/mL yielding a sensitivity of 75.5%, specificity of 82.2%, positive predictive value (PPV) of 86.3%, negative predictive value (NPV) of 69.3%, and overall accuracy of 78.2%; see Figure 4.

**Figure 5:**
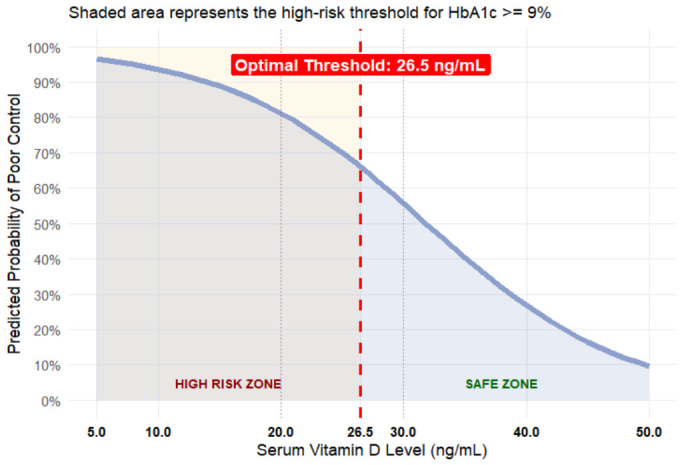
Probability nomogram for individualized risk assessment based on 25-hydroxyvitamin D levels.

The risk nomogram showed a steep inverse sigmoidal relationship. Patients with 25-hydroxyvitamin D levels < 10 ng/mL had a predicted probability of poor control exceeding 90%, while levels > 40 ng/mL were associated with a risk of less than 25%; see Figure 5.

**Figure 6:**
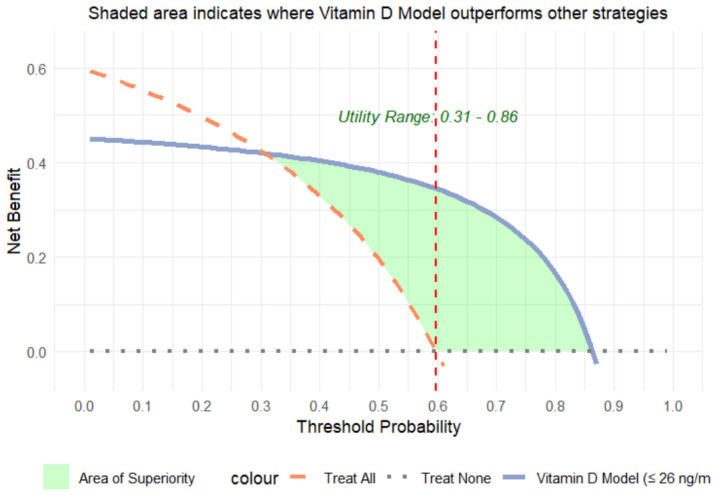
Decision Curve Analysis (DCA) demonstrates the net clinical benefit of 25-hydroxyvitamin D testing. The *y*-axis represents Net Benefit, while the *x*-axis indicates the Threshold Probability. The 25-hydroxyvitamin D Model (solid blue line) demonstrates superior potential clinical utility compared to the ‘Treat-All’ strategy (orange dashed line) across a threshold probability range of approximately 32% to 85%. Within this shaded ‘Area of Superiority,’ the model provides the highest net benefit, effectively identifying patients at risk for poor glycemic control while minimizing unnecessary interventions. Below a 32% threshold, the ‘Treat-All’ strategy remains optimal, while the ‘Treat-None’ strategy (grey dotted line) serves as the baseline for zero net benefit.

DCA demonstrated that the model incorporating serum 25-hydroxyvitamin D levels provided superior net benefit compared with both the “treat-all” and “treat-none” strategies across a threshold probability range of 32–85%. For clinicians with a risk threshold in this range, the 25-hydroxyvitamin D model offered the highest net benefit by effectively identifying patients at risk of poor glycemic control while minimizing unnecessary interventions. Below a threshold of 32%, the “treat-all” strategy remained the most beneficial approach, while beyond a threshold of 85%, the model’s net benefit diminished to zero; see Figure 6.

## 4. Discussion

This cross-sectional study of children and adolescents with T1DM revealed a high prevalence of suboptimal vitamin D status, affecting nearly two-thirds of participants. The most important finding was a strong, independent, and inverse association between 25-hydroxyvitamin D levels and glycemic control.

In the current study, 64.3% had insufficient or deficient vitamin D levels. Similar high prevalence was reported in United States [vitamin D deficiency (4%) and vitamin D insufficiency (60%)] [31]. A higher prevalence was reported in Switzerland [deficiency (60.5%), insufficiency (26.4%)], Bangladish [deficiency (51.7%) and insufficiency (23.3%)] [32], and Iran [deficiency (77%) and insufficiency (23%)] [33]. On the other hand, a lower prevalence of 45% (95% confidence interval [CI] 37–54%, *I*^2^ = 97.94%) was reported in a meta-analysis of 45 studies involving 6995 participants [34]. Also, a lower prevalence was reported in China (49.7) [35] and Turkey (38%) [36]. The high prevalence of vitamin D deficiency in this population may be attributed to dark skin pigmentation in the southern region of Saudi Arabia. Increased melanin content reduces the penetration of UV light into deeper layers of the skin, thereby impairing cutaneous vitamin D synthesis. However, genetic causes, such as vitamin D receptor gene polymorphisms, may represent an additional contributing factor [37]. Moreover, vitamin D intake in this study was assessed exclusively from dietary sources, as we did not enroll patients with a history of vitamin D supplementation. In addition, food sources of vitamin D are very limited unless they are fortified [38]. It is worth noting that the association between T1DM and vitamin D deficiency remains a subject of ongoing debate. A systematic review and meta-analysis concluded that insufficiency or deficiency of vitamin D is associated with the development of T1DM in children [39], while evidence from observational studies suggests that vitamin D supplementation during infancy may be protective against the development of T1DM [40].

Participants with new-onset T1DM had a lower prevalence of vitamin D deficiency and insufficiency compared with those with established disease; however, this difference was not statistically significant, consistent with findings reported by Liu et al. [35], who observed higher mean vitamin D levels in newly diagnosed children with T1DM compared with those with established disease, without reaching statistical significance.

In this study, vitamin D deficiency and insufficiency were more prevalent among females compared with males. Likewise, several studies reported similar findings [37,41]. This pattern may reflect cultural and religious practices in Saudi Arabia, where female dress conventions including head and body covering limit sun exposure and subsequent vitamin D synthesis. Additionally, females may have relatively higher vitamin D requirements during the pubertal growth spurt to support the demands of rapid bone growth and mineralization [38].

In this study, there was a statistically significant difference in the prevalence of vitamin D deficiency across different age groups. It is important to highlight that multiple studies have shown that older children tend to have a higher prevalence of vitamin D deficiency, which has been linked to reduced sunlight exposure, rapid growth spurts, and lower consumption of vitamin D-fortified foods [32,42]. We found a significant association between diabetes duration and weight with vitamin D deficiency. Likewise, other studies have reported a significant association [16,43].

Vitamin D and glycemic control: in this study we found significant association between 25-hydroxyvitamin D and HbA1c. Although conventional metrics such as AUC confirm the diagnostic accuracy of 25-hydroxyvitamin D, the DCA results highlights its real-world clinical usefulness in pediatric care. For a clinician whose risk threshold for poor control lies between 32% and 85%, a typical range for initiating intensive education or supplementation, the 25-hydroxyvitamin D model delivers a greater net benefit than a strategy of empirically treating all patients. These findings suggest that 25-hydroxyvitamin D functions not only as a general health marker but also as a potential risk stratification tool that warrants further investigation to optimize T1DM management. Similarly, Almansour et al. [44] also reported a significant association between vitamin D deficiency and uncontrolled T1DM in a Saudi pediatric population. On the other hand, a study conducted by Mutlu et al. [36] did not find a significant correlation between serum 25-hydroxyvitamin D levels and duration of diabetes or HbA1c. Several mechanisms could explain the effect of 25-hydroxyvitamin D on glycemic control. It influences glucose metabolism and glycemic control by suppressing inflammatory and autoimmune responses, facilitating insulin synthesis and secretion, improving insulin sensitivity, and interacting with vitamin D-related gene polymorphisms [45]. Furthermore, vitamin D contributes to the regulation of T-cell responses, thereby protecting pancreatic β-cells from immune-mediated destruction [46]. Vitamin D may also attenuate oxidative stress by reducing reactive oxygen species (ROS) production while regenerating glutathione (GSH), with the active metabolite calcitriol playing a prominent role in this antioxidant pathway [47]. Finally, vitamin D is believed to exert protective effects against diabetic complications, including nephropathy, retinopathy, and cardiovascular disease [48]. Nevertheless, while our findings demonstrate a strong association, they do not establish causality, and reverse causality cannot be excluded.

These findings carry important clinical implications. The high prevalence of suboptimal vitamin D status supports routine screening as standard care for all children and adolescents with T1DM. The strong association with HbA1c suggests that 25-hydroxyvitamin D management may serve as an adjunctive strategy to support glycemic control. Clinicians should prioritize screening high-risk groups and those with poor glycemic control.

### Strengths and Limitations

This study has several strengths. First, standardized definitions for 25-hydroxyvitamin D and glycemic control, based on Endocrine Society and ADA guidelines, enhance comparability with other studies. Second, the relatively large sample of children and adolescents with T1DM yields reliable estimates of vitamin D deficiency prevalence. Third, comprehensive demographic, clinical, anthropometric, and lifestyle data enabled thorough participant characterization. Fourth, appropriate non-parametric methods (Mann–Whitney U test, Spearman correlation) for non-normally distributed data strengthen the validity of the findings. Finally, decision curve analysis offers clinically meaningful insights beyond traditional statistical significance. This study has several important limitations. Dietary assessment represents a key methodological limitation. Nutritional intake was captured in the electronic medical record using a single self-reported binary variable (“balanced diet: yes/no”), which precludes evaluation of total caloric intake, vitamin D intake, fortified food consumption, or overall diet quality. Sunlight exposure, outdoor physical activity, and socioeconomic status were also not assessed, all of which may influence vitamin D levels. Future studies should incorporate validated dietary assessment tools (e.g., food frequency questionnaires or 24-h dietary recalls) and systematically evaluate sun exposure and socioeconomic factors. In addition, the cross-sectional design limits causal inference. Although an association was observed between 25-hydroxyvitamin D levels and glycemic control, reverse causality is plausible. Poor glycemic control may reduce 25-hydroxyvitamin D levels through inflammation, reduced outdoor activity, altered dietary intake, metabolic changes, and associated comorbidities. Prospective cohort studies and randomized controlled trials are needed to clarify directionality. An additional limitation is potential confounding by comorbid autoimmune and chronic conditions commonly associated with T1DM, including celiac disease, autoimmune thyroid disease, and chronic kidney disease. These conditions may independently lower 25-hydroxyvitamin D levels and influence glycemic control. Because they were not systematically screened or adjusted for, residual confounding is possible. Future studies should incorporate routine assessment of these comorbidities to better isolate the relationship between vitamin D status and glycemic control.

## 5. Conclusions and Recommendations

This study demonstrates a strikingly high prevalence of vitamin D deficiency and insufficiency among children and adolescents with T1DM. Furthermore, it demonstrates a strong, inverse association between vitamin D deficiency levels and HbA1c, with vitamin D sufficiency being significantly more prevalent among patients who achieved optimal glycemic control. Based on these findings, we recommend 25-hydroxyvitamin D screening for all pediatric patients with T1DM. For those identified as deficient or insufficient, supplementation should be considered, not only for its well-established skeletal benefits but also as a potential adjunctive strategy to support metabolic control. Future prospective cohort studies and randomized controlled trials are warranted to determine whether early and sustained correction of vitamin D deficiency can improve long-term glycemic outcomes and reduce the risk of diabetes-related complications in this vulnerable population.

## Figures and Tables

**Table 1:** Descriptive Characteristics of Study Participants (*N* = 266).

Variable	Category	Value
Age	Years, median (IQR)	13.0 [10.0–15.0]
	0–4 years, *n* (%)	14 (5.3)
	5–9 years, *n* (%)	51 (19.2)
	10–14 years, *n* (%)	107 (40.2)
	15–18 years, *n* (%)	94 (35.3)
Sex	Male, *n* (%)	151 (56.8)
	Female, *n* (%)	115 (43.2)
Diabetes duration	Years, median (IQR)	2.0 [1.0–4.0]
	New onset, *n* (%)	87 (32.7)
	Intermediate, *n* (%)	163 (61.3)
	Long-standing, *n* (%)	16 (6.0)
Glycemic control	HbA1c, %, median (IQR)	9.7 [7.8–11.4]
	Optimal, *n* (%)	31 (11.7)
	Acceptable, *n* (%)	24 (9.0)
	Suboptimal, *n* (%)	52 (19.5)
	Poor, *n* (%)	159 (59.8)
Time in range	Median [IQR]	40% [30–70]
	Good, *n* (%)	66 (24.8)
	Fair, *n* (%)	41 (15.4)
	Poor, *n* (%)	159 (59.8)
BMI	kg/m^2^, median [IQR]	18.9 [17.0–22.3]
	Underweight, *n* (%)	10 (3.8)
	Normal weight, *n* (%)	180 (67.7)
	Overweight, *n* (%)	46 (17.3)
	Obese, *n* (%)	30 (11.3)

Values are presented as median [IQR] or *n* (%). IQR = interquartile range; HbA1c = glycated hemoglobin; BMI = body mass index.

**Table 2:** Association between 25-hydroxyvitamin D Status and demographic, clinical, and anthropometric characteristics in T1DM Patients (*N* = 266).

Characteristic	Category	Suboptimal (Deficient/Insufficient) (*n* = 171)	Sufficient (*n* = 95)	*p* Value
Gender	Female	81 (70.4%)	34 (29.6%)	0.090
	Male	90 (59.6%)	61 (40.4%)	
Age group ^+^	0–4 years	10 (71.4%)	4 (28.6%)	0.032
	5–9 years	27 (52.9%)	24 (47.1%)	
	10–14 years	79 (73.8%)	28 (26.2%)	
	15–18 years	55 (58.5%)	39 (41.5%)	
Diabetes onset	below 5 years	19 (63.3%)	11 (36.7%)	0.280
	5–10 years	87 (69.0%)	39 (31%)	
	Above 10 years	65 (59.1%)	45 (40.9%)	
BMI category	Underweight	7 (70.0%)	3 (30.0%)	0.2221
	Normal weight	122 (67.8%)	58 (32.2%)	
	Obese	15 (50.0%)	15 (50.0%)	
	Overweight	27 (58.7%)	19 (41.3%)	
Height status	Normal stature	140 (62.2%)	85 (37.8%)	0.231
	Short stature	27 (90.0%)	3 (10.0%)	
	Tall stature	4 (66.7%)	2 (33.3%)	
Weight status	Normal weight	162 (66.7%)	81 (33.3%)	0.001
	Overweight/Obese	1 (11.1%)	8 (88.9%)	
	Underweight	8 (57.1%)	6 (42.9%)	
Diabetes duration	Intermediate	108 (66.3%)	55 (33.7%)	0.695
	Long-standing	10 (62.5%)	6 (37.5%)	
	New onset	53 (60.9%)	34 (39.1%)	
Balanced diet	No	31 (86.1%)	5 (13.9%)	0.006
	Yes	140 (60.9%)	90 (39.1%)	

*p* values were calculated using the Chi-square test (or Fisher’s exact test where appropriate). *p*-values (<0.05) indicate statistical significance. Balanced diet was defined as a self-reported dietary pattern meeting age-appropriate nutritional recommendation. ^+^ Although the overall association between age group and vitamin D status was statistically significant (*p* = 0.032), none of the pairwise comparisons remained significant after Bonferroni correction. The largest difference was observed between the 5–9 years and 10–14 years groups, but this did not retain significance after adjustment for multiple comparisons (adjusted *p* = 0.054).

**Table 3:** Comparison of clinical, demographic, and lifestyle characteristics between controlled and poorly controlled diabetes groups.

Category	Variable	HbA1c < 9% (*n* = 107)	HbA1c ≥ 9.0% (*n* = 159)	*p* Value
Demographics	Age, years [median, IQR]	13.0 [10.0–15.0]	13.0 [9.5–15.0]	0.507
	Age at onset, years [median, IQR]	10 [7–12]	10 [6.5–12]	0.458
	Sex, Female, *n* (%)	40 (34.8%)	75 (65.2%)	0.146
	Sex, Male, *n* (%)	67 (44.4%)	84 (55.6%)	
Diabetes duration	Duration, years [median, IQR]	2.0 [1.0–4.0]	2.0 [1.0–3.0]	0.714
	New onset, *n* (%)	35 (40.2%)	52 (59.8%)	0.956
	Intermediate, *n* (%)	65 (39.9%)	98 (60.1%)	
	Long-standing, *n* (%)	7 (43.8%)	9 (56.2%)	
Vitamin D status	25-hydroxyvitamin D, ng/mL [median, IQR]	35 [28–45]	21 [17–26]	<0.001
	Deficient, *n* (%)	5 (6.2%)	75 (93.8%)	<0.001
	Insufficient, *n* (%)	32 (35.2%)	59 (64.8%)	
	Sufficient, *n* (%)	70 (73.7%)	25 (26.3%)	
BMI status	BMI, kg/m^2^ [median, IQR]	19.0 [17.6–22.5]	18.3 [16–22]	0.198
	Underweight, *n* (%)	4 (40.0%)	6 (60.0%)	0.706
	Normal weight, *n* (%)	70 (38.9%)	110 (61.1%)	
	Overweight, *n* (%)	22 (47.8%)	24 (52.2%)	
	Obese, *n* (%)	11 (36.7%)	19 (63.3%)	
Lifestyle factors	Balanced diet, yes, *n* (%)	104 (45.2%)	126 (54.8%)	<0.001
	Balanced diet, No, *n* (%)	3 (8.3%)	33 (91.7%)	

*p* values calculated using the Chi-square test for categorical variables and the Mann–Whitney U test for continuous variables. Percentages may not sum to 100% due to rounding.

## Data Availability

The original data presented in the study are openly available in Data (https://alexuuni-my.sharepoint.com/:x:/g/personal/ramy_ghazy_alexu_edu_eg/IQBrgpQ7ofv5TZIBOfDZT_TYAXwLGrANmpxdqoSBS2kP_pY?e=Hgulvr&wdLOR=c3011FA01-2B28-42E1-A2E1-256BC890547A).

## Abbreviations

ADA	American Diabetes Association
AUC	Area Under the Curve
BMI	Body Mass Index
CDC	Centers for Disease Control and Prevention
CI	Confidence Interval
DCA	Decision Curve Analysis
DKD	Diabetic Kidney Disease
GSH	Glutathione
HbA1c	Glycated Hemoglobin
IQR	Interquartile Range
IRB	Institutional Review Board
NPV	Negative Predictive Value
OR	Odds Ratio
PPV	Positive Predictive Value
ROC	Receiver Operating Characteristic
ROS	Reactive Oxygen Species
SD	Standard Deviation

## References

[1] Youness R.A., Dawoud A., ElTahtawy O., Farag M.A. (2022). Fat-soluble vitamins: Updated review of their role and orchestration in human nutrition throughout life cycle with sex differences. Nutr. Metab..

[2] Deepika, Kumari A., Singh S., Ahmad M.F., Chaki D., Poria V., Kumar S., Saini N., Yadav N., Sangwan N. (2025). Vitamin D: Recent advances, associated factors, and its role in combating non-communicable diseases. npj Sci. Food.

[3] Wacker M., Holick M.F. (2013). Sunlight and Vitamin D: A global perspective for health. Derm.-Endocrinol..

[4] de Bock M., Codner E., Craig M.E., Huynh T., Maahs D.M., Mahmud F.H., Marcovecchio L., DiMeglio L.A. (2022). ISPAD Clinical Practice Consensus Guidelines 2022: Glycemic targets and glucose monitoring for children, adolescents, and young people with diabetes. Pediatr. Diabetes.

[5] Mostafa W.Z., Hegazy R.A. (2015). Vitamin D and the skin: Focus on a complex relationship: A review. J. Adv. Res..

[6] Grant W.B., Wimalawansa S.J., Pludowski P., Cheng R.Z. (2025). Vitamin D: Evidence-Based Health Benefits and Recommendations for Population Guidelines. Nutrients.

[7] Zittermann A., Prokop S. (2014). The role of vitamin D for cardiovascular disease and overall mortality. Adv. Exp. Med. Biol..

[8] Liu Y., Yu Q., Zhu Z., Zhang J., Chen M., Tang P., Li K. (2015). Vitamin and multiple-vitamin supplement intake and incidence of colorectal cancer: A meta-analysis of cohort studies. Med. Oncol..

[9] Bergman P., Lindh A.U., Björkhem-Bergman L., Lindh J.D. (2013). Vitamin D and Respiratory Tract Infections: A Systematic Review and Meta-Analysis of Randomized Controlled Trials. PLoS ONE.

[10] Aribi M., Mennechet F.J.D., Touil-Boukoffa C. (2023). Editorial: The role of vitamin D as an immunomodulator. Front. Immunol..

[11] Cui A., Zhang T., Xiao P., Fan Z., Wang H., Zhuang Y. (2023). Global and regional prevalence of vitamin D deficiency in population-based studies from 2000 to 2022: A pooled analysis of 7.9 million participants. Front. Nutr..

[12] Ghazy R.M., Alsaleem S.A., Alshaikh A.A., Al-Qahtani F.S., Shehata S.F., AlHefdhi H.A., Habbash A.S., Alhumayed R.S., Alsamghan A. (2026). Epidemiological Transition and Forecasting of Diabetes Burden in Saudi Arabia: A Comprehensive Analysis From the Global Burden of Disease Study 1990–2023. Diabetes Obes. Metab..

[13] Monteiro L.E.d.R.C., Garcia S.P., Bottino L.G., Custodio J.L., Telo G.H., Schaan B.D. (2022). Precipitating factors of diabetic ketoacidosis in type 1 diabetes patients at a tertiary hospital: A cross-sectional study with a two-time-period comparison. Arch. Endocrinol. Metab..

[14] Shen L., Zhuang Q.S., Ji H.F. (2016). Assessment of vitamin D levels in type 1 and type 2 diabetes patients: Results from metaanalysis. Mol. Nutr. Food Res..

[15] Jacobsen R., Frederiksen P., Heitmann B.L. (2016). Exposure to sunshine early in life prevented development of type 1 diabetes in Danish boys. J. Pediatr. Endocrinol. Metab..

[16] Svoren B.M., Volkening L.K., Wood J.R., Laffel L.M. (2009). Significant vitamin D deficiency in youth with type 1 diabetes mellitus. J. Pediatr..

[17] Janner M., Ballinari P., Mullis P.E., Flück C.E. (2010). High prevalence of vitamin D deficiency in children and adolescents with type 1 diabetes. Swiss Med. Wkly..

[18] Barot K.S., Abbasi Z.A., Krishna Mohan G.V., Abid S.A., Hussain S.A., Wei C.R., Ali N. (2025). Prevalence of Vitamin D Deficiency in Children and Adolescents with Type 1 Diabetes Mellitus: A Systematic Review and Meta-Analysis. Cureus.

[19] Lopes M., Laiginhas R., Madeira C., Neves J.S., Barbosa M., Rosas V., Carvalho D., Falcão-Reis F., Falcão M. (2020). Association between Serum Vitamin D and Diabetic Retinopathy in Portuguese Patients with Type 1 Diabetes. Acta Med. Port..

[20] Asmaa Saeed B., Nahla Abd El-Aziz N., Abeer Mohamed S. (2023). Assessment of serum vitamin D level in children with type 1 diabetes mellitus: A cross-sectional study. J. Pak. Med. Assoc..

[21] Wu J., Atkins A., Downes M., Wei Z. (2023). Vitamin D in diabetes: Uncovering the sunshine hormone’s role in glucose metabolism and beyond. Nutrients.

[22] Cutolo M., Plebani M., Shoenfeld Y., Adorini L., Tincani A. (2011). Vitamin D endocrine system and the immune response in rheumatic diseases. Vitam. Horm..

[23] Gembillo G., Cernaro V., Salvo A., Siligato R., Laudani A., Buemi M., Santoro D. (2019). Role of Vitamin D Status in Diabetic Patients with Renal Disease. Medicina.

[24] Gembillo G., Siligato R., Amatruda M., Conti G., Santoro D. (2021). Vitamin D and Glomerulonephritis. Medicina.

[25] Figueiredo Moreira C.F., Ferreira Peres W.A., Silva do Nascimento Braga J., Proença da Fonseca A.C., Junior M.C., Luescher J., Campos L., de Carvalho Padilha P. (2025). Effect of vitamin D supplementation on glycemic control in children and adolescents with type 1 diabetes mellitus: Data from a controlled clinical trial. Diabetes Res. Clin. Pract..

[26] Duncan B.B., Magliano D.J., Boyko E.J. (2026). IDF diabetes atlas 11th edition 2025: Global prevalence and projections for 2050. Nephrol. Dial. Transplant..

[27] Saggese G., Vierucci F., Prodam F., Cardinale F., Cetin I., Chiappini E., De’ Angelis G.L., Massari M., Miraglia Del Giudice E., Miraglia Del Giudice M. (2018). Vitamin D in pediatric age: Consensus of the Italian Pediatric Society and the Italian Society of Preventive and Social Pediatrics, jointly with the Italian Federation of Pediatricians. Ital. J. Pediatr..

[28] American Diabetes Association Professional Practice Committee for Diabetes (2025). 14. Children and Adolescents: Standards of Care in Diabetes—2026. Diabetes Care.

[29] Danne T., Nimri R., Battelino T., Bergenstal R.M., Close K.L., DeVries J.H., Garg S., Heinemann L., Hirsch I., Amiel S.A. (2017). International Consensus on Use of Continuous Glucose Monitoring. Diabetes Care.

[30] El Mouzan M., Al Salloum A., Al Omer A., Alqurashi M., Al Herbish A. (2016). Growth reference for Saudi school-age children and adolescents: LMS parameters and percentiles. Ann. Saudi Med..

[31] Carakushansky M., Patel P., Ben Khallouq B.A., Gurnurkar S. (2020). Prevalence of Vitamin D Deficiency in Children with Type 1 Diabetes Mellitus. Cureus.

[32] Zabeen B., Nahar J., Ahmed B., Tayyeb S., Islam N., Azad K. (2022). Vitamin D status in children and adolescents with type 1 diabetes in a specialized diabetes care centre in Bangladesh. Endocrinol. Diabetes Metab..

[33] Ataie-Jafari A., Rahmat A.B., Abbasi F., Cheong Loke S., Qorbani M., Larijani B. (2012). Vitamin D status and associated factors in recent-onset type 1 diabetic children in Iran. J. Diabetes Metab. Disord..

[34] Yang X., Chai M., Lin M. (2024). Proportion of vitamin D deficiency in children/adolescents with type 1 diabetes: A systematic review and meta-analysis. BMC Pediatr..

[35] Liu C., Wang J., Wan Y., Xia X., Pan J., Gu W., Li M. (2018). Serum vitamin D deficiency in children and adolescents is associated with type 1 diabetes mellitus. Endocr. Connect..

[36] Mutlu A., Mutlu G.Y., Özsu E., Çizmecioğlu F.M., Hatun Ş. (2011). Vitamin D deficiency in children and adolescents with type 1 diabetes. J. Clin. Res. Pediatr. Endocrinol..

[37] Hamed E.O., Abdel-Aal A.M., Din A.K., Atia M.M. (2013). Vitamin D level and Fok-I vitamin D receptor gene polymorphism in Egyptian patients with type-1 diabetes. Egypt. J. Immunol..

[38] Razzaghy-Azar M., Shakiba M. (2010). Assessment of vitamin D status in healthy children and adolescents living in Tehran and its relation to iPTH, gender, weight and height. Ann. Hum. Biol..

[39] Liu C., Lu M., Xia X., Wang J., Wan Y., He L., Li M. (2015). Correlation of serum vitamin d level with Type 1 diabetes mellitus in children: A meta-analysis. Nutr. Hosp..

[40] Zipitis C.S., Akobeng A.K. (2008). Vitamin D supplementation in early childhood and risk of type 1 diabetes: A systematic review and meta-analysis. Arch. Dis. Child..

[41] Isa H., Almaliki M., Alsabea A., Mohamed A. (2020). Vitamin D deficiency in healthy children in Bahrain: Do gender and age matter?. East Mediterr. Health J..

[42] Roh Y.E., Kim B.R., Choi W.B., Kim Y.M., Cho M.J., Kim H.Y., Park K.H., Kim K.H., Chun P., Kim S.Y. (2016). Vitamin D deficiency in children aged 6 to 12 years: Single center’s experience in Busan. Ann. Pediatr. Endocrinol. Metab..

[43] Vojtková J., Ciljaková M., Vojarová L., Janíková K., Michnová Z., Sagiová V. (2012). Hypovitaminosis D in children with type 1 diabetes mellitus and its influence on biochemical and densitometric parameters. Acta Medica.

[44] Almansour S., Alsalamah A., Almutlaq M., Sheikh A., Hamdan H.Z., Al-Nafeesah A., AlEed A., Adam I., Al-Wutayd O. (2024). Association of vitamin D deficiency and insufficiency with uncontrolled type 1 diabetes Mellitus among Saudi pediatric patients; a hospital-based retrospective study. Front. Pediatr..

[45] Szodoray P., Horvath I.F., Papp G., Barath S., Gyimesi E., Csathy L., Kappelmayer J., Sipka S., Duttaroy A.K., Nakken B. (2010). The immunoregulatory role of vitamins A, D and E in patients with primary Sjogren’s syndrome. Rheumatology.

[46] El-Fakhri N., McDevitt H., Shaikh M.G., Halsey C., Ahmed S.F. (2014). Vitamin D and its effects on glucose homeostasis, cardiovascular function and immune function. Horm. Res. Paediatr..

[47] Cetinkalp S., Delen Y., Karadeniz M., Yüce G., Yilmaz C. (2009). The effect of 1alpha,25(OH)2D3 vitamin over oxidative stress and biochemical parameters in rats where Type 1 diabetes is formed by streptozotocin. J. Diabetes Complicat..

[48] Musazadeh V., Kavyani Z., Mirhosseini N., Dehghan P., Vajdi M. (2023). Effect of vitamin D supplementation on type 2 diabetes biomarkers: An umbrella of interventional meta-analyses. Diabetol. Metab. Syndr..

